# Depression among caregivers in emergency and intensive care

**DOI:** 10.1192/j.eurpsy.2023.516

**Published:** 2023-07-19

**Authors:** N. Rmadi, I. Sellami, A. Feki, W. Falah, L. Ghanmi, M. L. Masmoudi, K. Jmal Hammami, M. Hajjaji

**Affiliations:** ^1^Department Of Occupational Medicine; ^2^Department of rheumatology, HEDI CHAKER hospital, University of Sfax; ^3^University of Sfax, SFAX, Tunisia

## Abstract

**Introduction:**

Working in intensive care units and in emergencies is a stressful job. Taking care of acute and serious pathologies may cause various psychological diseases.

**Objectives:**

This study aimed to screen depression among emergency and intensive care caregivers and to determine factors associated with these disorders.

**Methods:**

This is an exhaustive, descriptive and analytical cross-sectional study that interested paramedical caregivers working in the emergency and intensive care services from south Tunisia. We used an anonymous questionnaire that included sociodemographic, medical and professional characteristics and the subscale of depression from the Hospital anxiety and depression (HAD) scale.

**Results:**

A total of 240 patients participated in the survey. The prevalence of depression was 30.8%. In the univariate study, depression was associated with the female sex (P=0.006), university level (p=0.04) and anxiety (p<10-3). Three risk factors of depression were found in the multivariate analysis: female gender (OR=2.4 [1.1-7]; P=0.025), and the university school level (OR=5[1.5-16.7]; P =0.009).

**Conclusions:**

Depressive disorders are common among caregivers in emergency and intensive care units. This finding highlights the importance of an early screening of these disorders to improve their management.

**Disclosure of Interest:**

None Declared

